# Bacteriological quality of drinking water and its associated factors in Ethiopia: A systematic review and meta-analysis

**DOI:** 10.1371/journal.pone.0310731

**Published:** 2025-01-03

**Authors:** Chala Daba, Leykun Berhanu, Belay Desye, Gete Berihun, Abebe Kassa Geto

**Affiliations:** 1 Department of Environmental Health, College of Medicine and Health Sciences, Wollo University, Dessie, Ethiopia; 2 Dessie Health Science College, Dessie, Ethiopia; BHU: Bule Hora University, Institute of Health, ETHIOPIA

## Abstract

**Introduction:**

Drinking contaminated water is a significant cause of mortality and morbidity in Sub-Saharan Africa, where access to safe drinking water is limited. Although numerous studies have investigated the bacteriological quality of drinking water in Ethiopia, their findings have been inconsistent and varied, hindering the implementation of effective water quality monitoring. Moreover, there is a lack of nationwide assessment of the bacteriological quality of drinking water in Ethiopia. Therefore, this systematic review and meta-analysis aimed to determine the bacteriological quality of drinking water and its associated factors in Ethiopia.

**Methods:**

An international electronic database, including PubMed, Science Direct, Global Health, CINAHL, African Journals Online, HINARI, and Google Scholar was employed to retrieve the relevant articles. The study adhered to the Preferred Reporting Items for Systematic Reviews and Meta-Analyses Protocols (PRISMA) guidelines. A random-effects model was used to estimate the pooled effect size, and the Egger regression model was employed using STATA 14 software to assess potential publication bias.

**Results:**

A total of 26 studies involving 7,962 water samples met the eligibility criteria for meta-analysis. The pooled prevalence of at least one bacteriological contamination of drinking water was 52.26% (95%CI: 39.09–65.43), with extreme heterogeneity (I^2^ = 99.7%; p-value < 0.001). The pooled prevalence of total and fecal coliform in drinking water was 49.55% (95% CI: 34.88–64.23) and 44.27% (95%CI: 34.36–54.19), respectively. 14.13% of the water source was at a very high sanitary risk level (unfit for drinking), with significant heterogeneity (I^2^ = 94.1%, p< 0.001). The absence of household-level water treatment (OR = 3.3; 95%CI: 1.28–5.32) and drawing water using dipping methods (OR = 4.52; 95%CI: 1.71–7.34) were determinant factors for bacteriological contamination of drinking water.

**Conclusion:**

We found that the bacteriological quality of drinking water did not comply with the World Health Organization and Ethiopia’s standard guidelines for drinking water, which call for urgent intervention. One out of seven water sources was at a high sanitary risk level, which could increase the risk of infectious disease in the country. The absence of household-level water treatment and drawing water using dipping was a significant factor in the bacteriological quality of drinking water. Based on these findings, the water supply and sewerage authority should prioritize regular monitoring of the bacteriological quality of drinking water.

## Introduction

Access to safe drinking water is one of the fundamental human rights and it’s the top priority of governments in all nations, which is stated in the global agenda of the Sustainable Development Goals (SDGs) 6 [1]. However, more than 785 million people worldwide use unimproved water sources and two billion people use drinking water contaminated with feces [2], which indicates drinking contaminated water is a persistent public health problem across the global communities particularly in developing countries. World Health Organization (WHO) report of 2019 also indicates that around 80% of global illnesses and diseases were mainly due to water-related diseases [2]. Drinking contaminated water serves as a mechanism to transmit communicable diseases such as diarrhea, dysentery and cholera [3]. According to a recent report by WHO, around 1.6 million deaths were reported due to a lack of safe water, sanitation, and hygiene [4]. A recent global burden of disease revealed that in 2019, 1.7 million deaths were linked to diarrhea and acute respiratory infections caused by inadequate water, sanitation, and hygiene (WASH) conditions [5].

Waterborne diseases are significant causes of premature morbidity and mortality, where the burden of the problem is high in low resource-limited countries [6]. Africa, particularly Sub-Saharan Africa, faces a severe public health crisis due to contaminated water. Over half of the population in Sub-Sahaan Africa including Ethiopia lacks access to safe drinking water sources, leading to a higher incidence of waterborne illnesses [7–9]. For instance, evidence from a recent report in Lesotho showed that 78% of unprotected water and 60% of protected water sources were contaminated with *Escherichia coli*, which could increase infectious diseases in the country [10]. Beyond mortality and morbidity, the problems associated with poor water quality have significant economic impacts and health expenses [11].

Contamination of drinking water is a persistent and growing problem in Ethiopia, posing a serious threat to public health. Evidence from a UNICEF report showed that 60–80% of infectious diseases in Ethiopia are linked to a lack of access to safe water, proper sanitation, and basic hygiene practices [12]. As a result of waterborne disease, more than 70,000 under-five children die a year, which calls for urgent interventions [12]. Various diseases in the communities are mainly due to ingestion of contaminated water [13]. While fecal contamination remains a significant source of drinking water contamination in Ethiopia, the problem is also expanding into piped water distribution systems, raising concerns about the safety of even treated water [14]. Evidence from different studies showed that frequent breakage in the distribution line [15], poor water handling practices [16], lack of knowledge [17], poor sanitation [18], inadequate fencing [19]), absence of household-level water treatment, and absence of hand washing practice before water collection [20] were the determinant factors for microbial contamination of drinking water.

Various investigations have been conducted to determine the bacteriological contamination of drinking water [15,16,18–34]. However, the evidence from these studies are inconclusive and characterized by significant variability [e.g., the prevalence of total coliform varied from 0.9% [35] -95.8% [15] and fecal coliform varied from 0.5% [35]- 84.4% [29]], which could negatively influence the design of effective water quality monitoring. The sanitary risk level of drinking water was not well investigated in the previous studies [15,19,22,27,35,36], which highlights the need for further studies. In addition, there is a lack of nationwide information about the bacteriological quality of drinking water and its associated factors in Ethiopia. Therefore, this meta-analysis aimed to assess the overall level of bacteriological contamination in drinking water in Ethiopia and pinpoint the factors that contribute to the bacteriological contamination. The results of this meta-analysis will provide valuable information for water quality monitoring efforts and the development of evidence-based interventions aimed at reducing the burden of waterborne diseases and related deaths in Ethiopia.

## Methods and materials

**Registration:** This systematic review and meta-analysis has been registered in PROSPERO under registration number CRD42024575421.

### Study selection, search strategy, and study period

The Preferred Reporting Items for Systematic Reviews and Meta-Analysis (PRISMA) guideline was used to conduct this meta-analysis [37] (S1 File). A comprehensive search of international electronic databases, including PubMed, HINARI, Global Health, Science Direct, African Journals Online, and Google Scholar, was conducted to retrieve relevant studies. The following key terms were used to search the studies: "bacteriological quality", "microbial quality", total coliform, fecal coliform, thermotolerant, "drinking water" "water", "water source", "protected water source", "unprotected water source", "tap", "factors", "associated factors", "determinant factors" and "Ethiopia". All key terms were combined using the Boolean operators “AND” or “OR” as appropriate (S2 File). The search was carried out up to August 10, 2024, by three authors independently (CD, BD, and LB).

### Inclusion and exclusion criteria

This meta-analysis focused on laboratory-based cross-sectional studies investigating the bacteriological quality of drinking water in Ethiopia. Studies published between 2000 and August 1, 2024, in the English language were considered for inclusion. However, studies that were unretrievable, qualitative in nature, had poor methodological quality or did not report the desired outcome were excluded from the analysis. Besides, conference papers, reviews and letters were excluded from the analysis.

### Outcome assessment

The primary outcome of this meta-analysis was to estimate the overall prevalence of bacteriological contamination in drinking water in Ethiopia. This was calculated by dividing the number of bacteriological contaminants by the total sample size and multiplying by 100. Additionally, the study aimed to identify factors associated with bacteriological contamination, expressed as a log odds ratio.

### Data extraction

All retrieved articles were imported into Endnote X20, and duplicates were removed. Three authors (CD, AG, and GB) independently extracted data using a standardized template. This template captured information such as author name, region, publication year, bacteriological detection methods, prevalence of total coliform, fecal coliform, E. coli, and sample size (S3 File).

### Quality assessment

After removing duplicate articles, three reviewers (CD, LB, and BD) screened the remaining articles for eligibility. The quality of each eligible article was then assessed by three reviewers (CD, GB, and BD) using the Joana Briggs Institute (JBI) critical appraisal checklist [38,39] (S4 File). Articles with a quality score above 50% on the JBI critical appraisal checklist were selected for further analysis [39,40]. If discrepancies arose during the quality assessment process, the mean score from all reviewers’ evaluations was calculated to resolve any differences.

### Statistical analysis and synthesis

A random-effects model was employed to calculate the pooled prevalence of bacteriological contamination in drinking water using the STATA 14 version [41]. Heterogeneity among the included studies was assessed using the Higgins I^2^ statistic, with values of 25%, 50%, and 75% representing low, moderate, and high heterogeneity, respectively [42]. A p-value below 0.05 indicated the presence of heterogeneity. Publication bias was investigated using a funnel plot and Egger’s test, with a p-value less than 0.05 suggesting publication bias [43].

Subgroup analysis was employed to pinpoint the source of publication bias based on various study characteristics such as the region where studies were conducted, sample size (large- ≥ 200) or small- < 200), and year of publication (2020 and after or before 2020). A sensitivity analysis was carried out to determine the effect of individual studies on the overall pooled prevalence estimates. Univariable meta-regression analysis was conducted to explore the relationship between the outcome variable (bacteriological contamination) and variables such as sample size, region, and year of publication. Additionally, a ’trim and fill’ analysis was performed to adjust the results and account for potential publication bias within the included studies.

## Results

### Study selection

**Fig 1:**
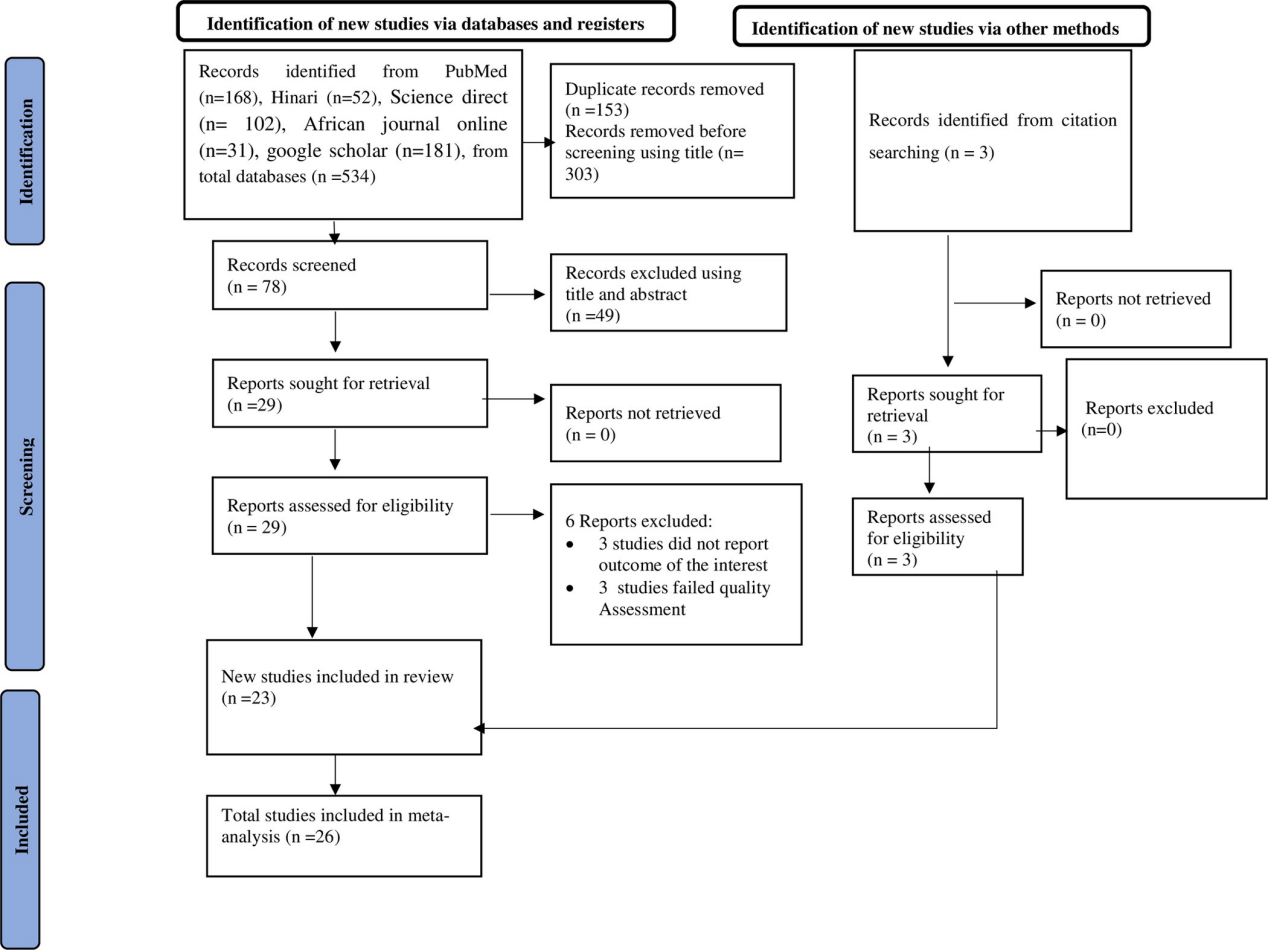
PRISMA flow diagram of the included studies for the systematic review and meta-analysis of bacteriological quality of drinking water and its associated factors in Ethiopia, 2024.

A total of 534 articles were identified from databases. Out of these, 49 studies were excluded based on their titles and abstracts. Using the Endnote reference manager, 456 duplicate studies were excluded. Moreover, 6 studies were excluded based on the quality assessment and outcomes of the interest (S2 File). Finally, 26 studies were eligible for this meta-systematic review and analysis **(Fig 1).**

### Characteristics of the included studies

A total of 26 articles [15,18–36,44–49] were included to determine the bacteriological quality of drinking water and its associated factors in Ethiopia. In this meta-analysis, a total of 7,962 of samples water were included. The highest prevalence of bacteriological contamination (total coliform) of drinking water was reported in studies conducted in the Amhara region (95.8%) [18] and the lowest prevalence of bacteriological contamination was observed in studies conducted in various regions (0.9%) [35]. Regarding the study region, ten studies were conducted in Amhara region [15,18,21,22,26,27,29,32,33,49], four in Oromia region [24,28,31,36], and two in Sidama [19,25], two in Addis Ababa [30,34], two in Tigray [47,48], one each in SNNPR [44], Dire Dawa [23], Somali [45], Harari [20], five regions [46] and various regions in Ethiopia [35] (Table 1).

**Table 1:** A descriptive summary of 26 studies included estimating the pooled prevalence of bacteriological quality of drinking water and its associated factors in Ethiopia, 2024.

Authors	Year of publication	Region	Type of water sample/source	Methods of data collection	Sample size/sample	Total coliform (%)	Fecal coliform (%)	*E*.*coli* (%)	Quality score (%)
Bedada et al [46]	2018	Five regions	Treated, untreated and bottled water	MPN	218	51.8	38.5	23.9	87.5
Wolde et al [34]	2020	Addis Ababa	Public taps, service reservoirs, springs and wells	Direct microscopy examination and P-A	2976	7	3	NA	75.0
Bedada et al [35]	2018	Various regions of Ethiopia	Bottled water	MPN	222	0.9	0.5	0.5	87.5
Sitotaw et al [15]	2021	Amhara	Reservoir, tap and HH container	MF	90	89	77	NA	75.0
Abera et al [22]	2014	Amhara	Spring reservoir and tap	MPN	140	21.4	18.6	17.8	75.0
Ashuro et al [19]	2021	Sidama	Dug well and spring	MF	213	NA	NA	50.2	75.0
Amenu et al [23]	2013	Dire Dawa	Well, spring and household tat	MF	90	90	75.5	NA	62.5
Gebrewahd et al [48]	2020	Tigray	Hand pump and spring	MPN	290	29	3.4	62.8	75.0
Sebsibe et al [31]	2021	Oromia	Tap and reservoir	MF	250	24.8	17.6	NA	75.0
Damtie et al [27]	2014	Amhara	Well, spring and tap	Rapid microbiological test strips	71	64.8	NA	NA	87.5
Admassu et al [49]	2004	Amhara	Spring, well and water lines	NR	70	35.7	25.7	22.8	87.5
Amenu et al [24]	2014	Oromia	Hand-dung well, borehole, spring, dugouts and Rainwater from the roof	MF	233	NA	NA	54.9	62.5
Sitotaw [18]	2021	Amhara	Borehole, reservoir, tap and container	MF	120	95.8	74.2	NA	75.0
Negera et al [36]	2017	Oromia	River, spring, well and hand pipe	MPN	93	75.2	65.5	61.3	62.5
Abera et al [21]	2016	Amhara	Pipeline, reservoir, spring and dug well	MPN	1030	44.7	27.9	29	75.0
Asefa et al [20]	2021	Harari	Deep well, hallow well, public tap	MF	448	NA	80.1	NA	75.0
Bekuretsion et al [47]	2018	Tigray	Hand pump borehole	MF	75	15	NA	4	87.5
Alemayehu et al [44]	2020	SNNPR	Boreholes, springs and wells	NR	340	NA	NA	44.7	87.5
Girmay et al [30]	2021	Addis Ababa	Water storage	MF	125	NA	NA	28.5	62.5
Tabor et al [32]	2011	Amhara	Tap and household water	MF	70	67.1	64.3	NA	75.0
Berhanu et al [25]	2015	Sidama	Spring and well	NR	21	NA	NA	NA	87.5
Eliku et al [28]	2015	Oromia	Tap water	MF	52	NA	NA	NA	75.0
Berihun et al [26]	2023	Amhara	Tap water	MF	412	NA	66	NA	75.0
Tsega et al [33]	2013	Amhara	Tap, protected and open dug well, spring	MF	53	83	35.8	NA	75.0
Feleke et al [29]	2018	Amhara	Stored drinking water	NR	135	NA	84.4	NA	75.0
Asfaw et al [45]	2016	Somali	Container, pipeline, water reservoir, ‘Beyollie’, and main sources	MPN	125	NA	NA	52	75.0

Hint: NA (Not available); MPN (Most Probable Number); MF (Membrane Filtration); NR (Not reported).

### Meta-analysis

**Fig 2:**
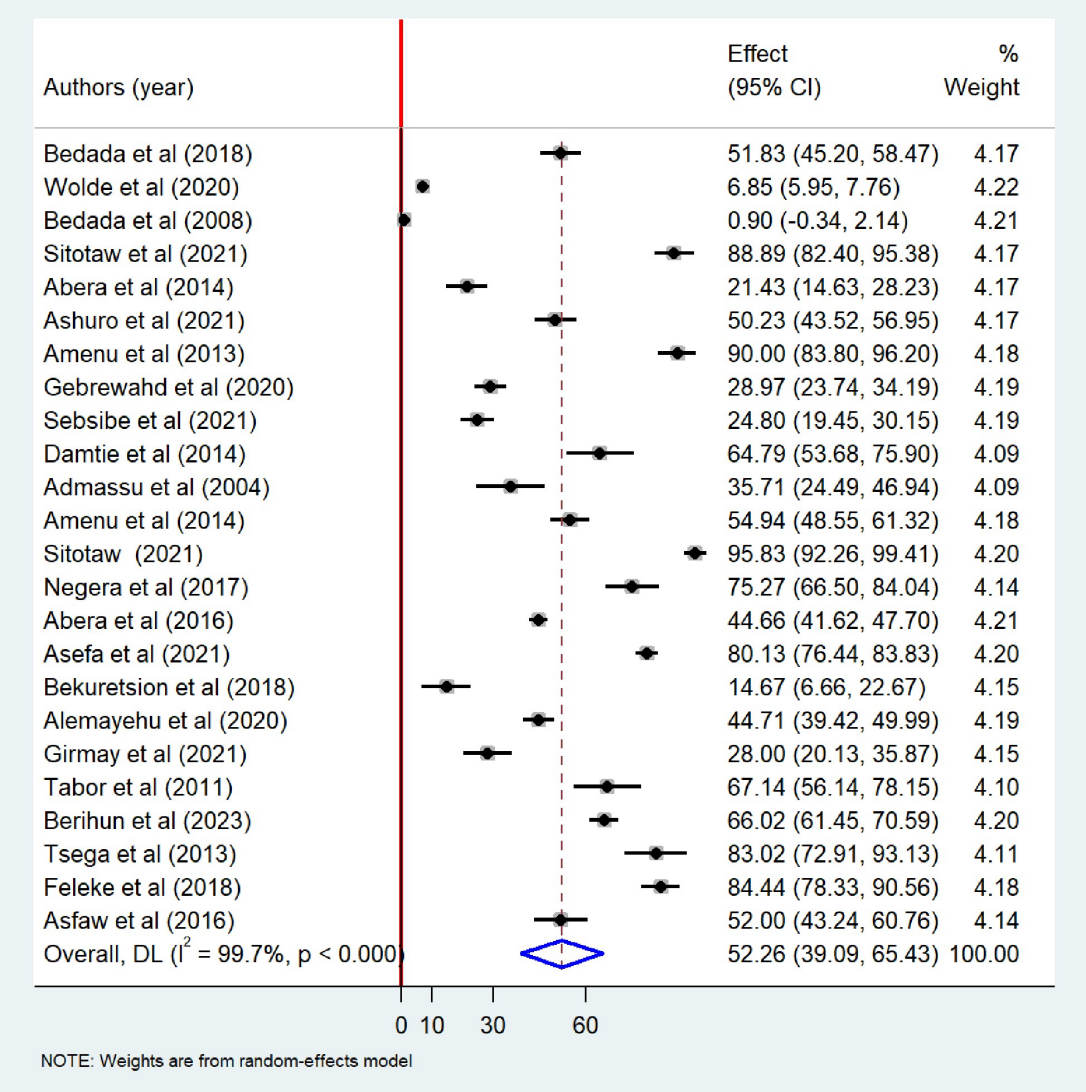
Forest plot showing at least one bacteriological contamination of drinking water in Ethiopia, 2024.

**Fig 3:**
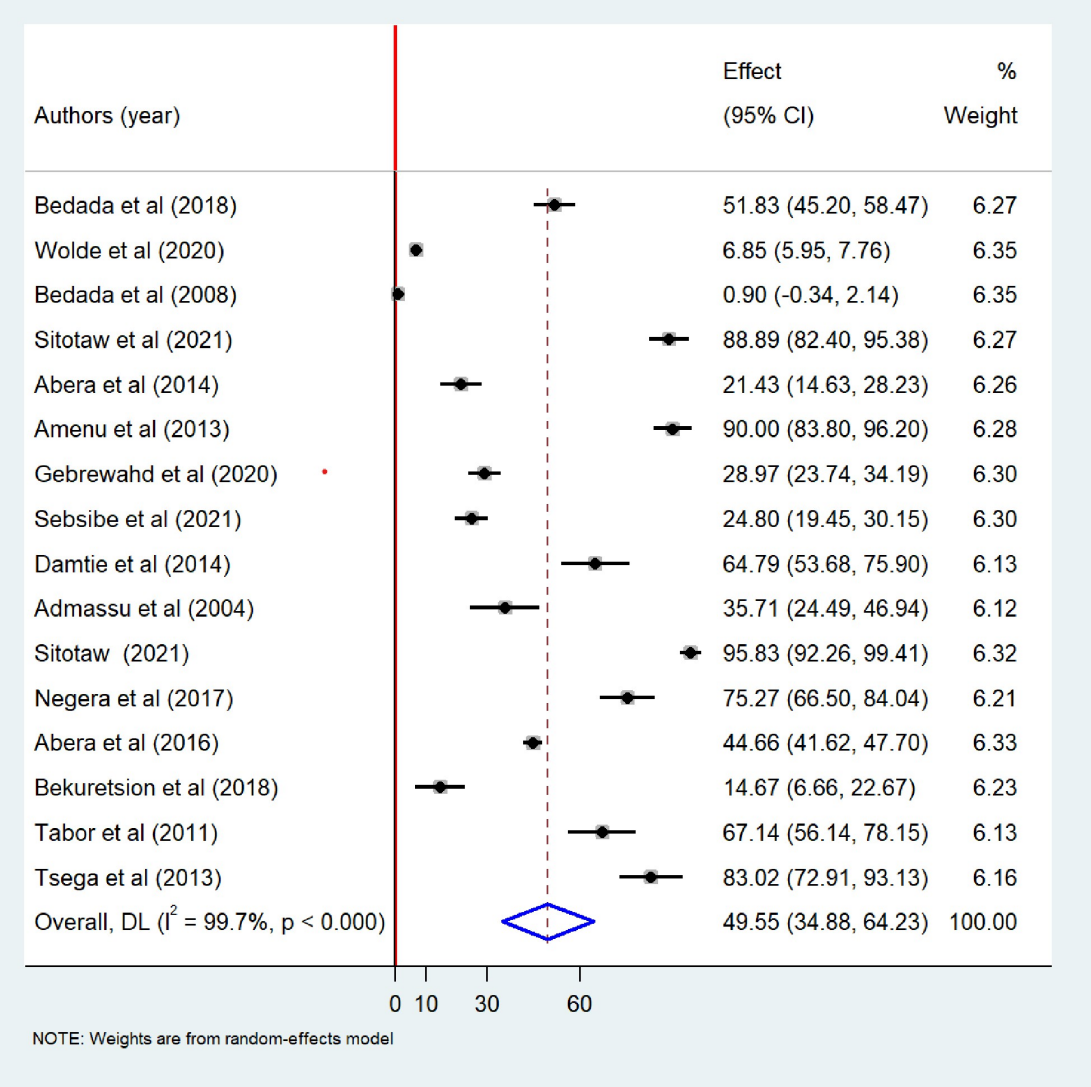
Forest plot showing total coliform contamination of drinking water in Ethiopia, 2024.

**Fig 4:**
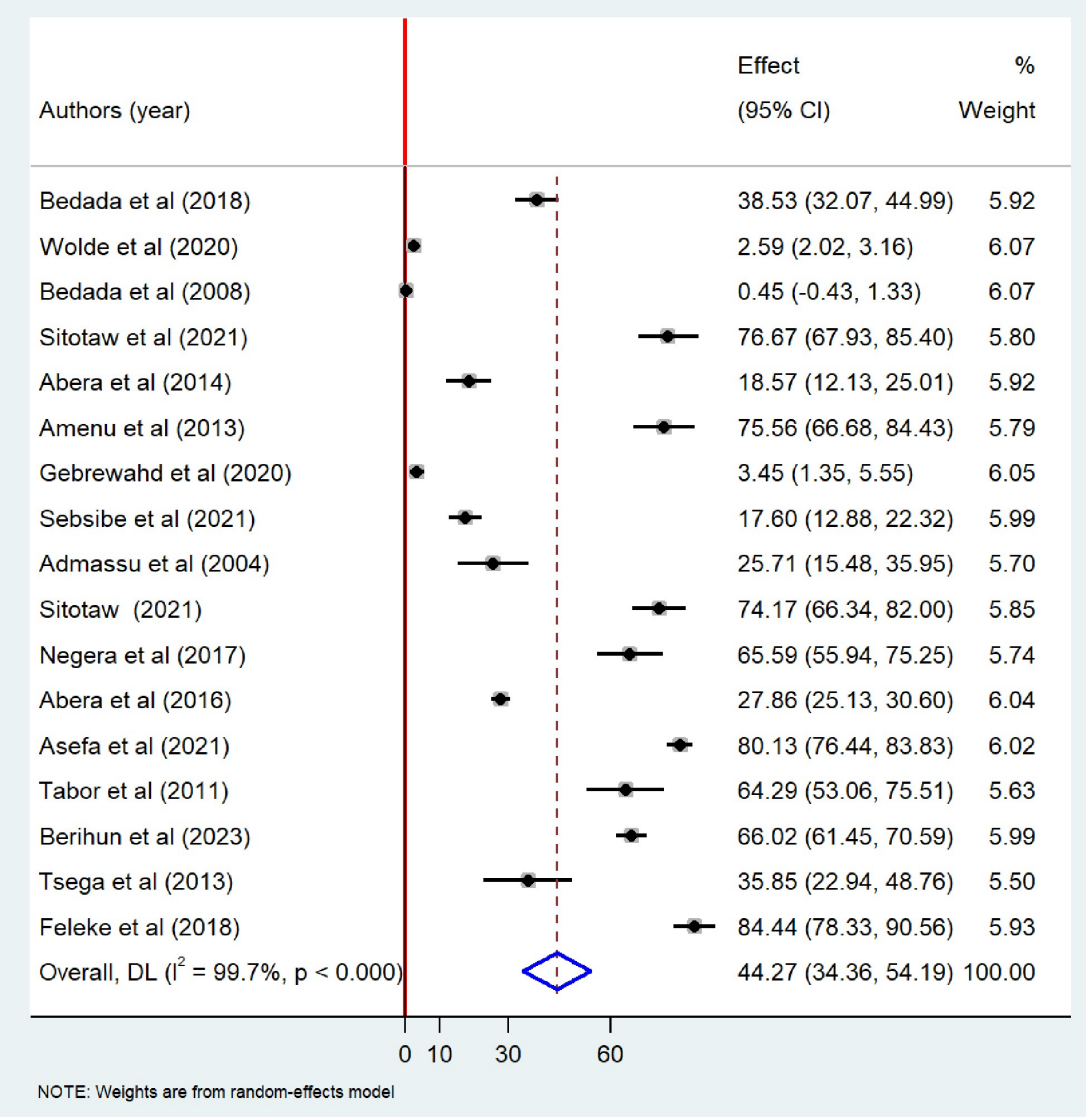
Forest plot showing fecal coliform contamination of drinking water in Ethiopia, 2024.

**Fig 5:**
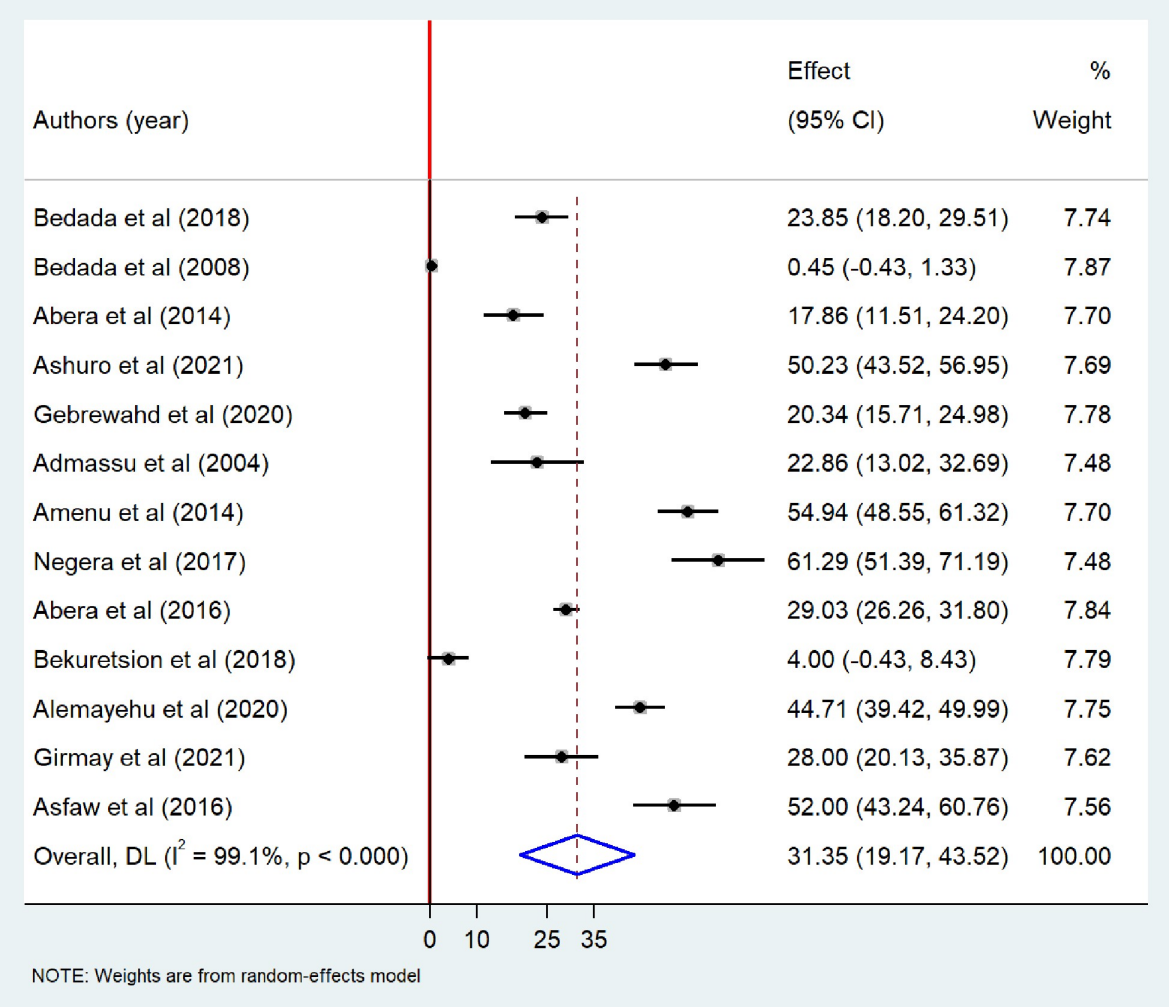
Forest plot showing E. coli contamination of drinking water in Ethiopia, 2024.

Meta-analysis revealed that the pooled prevalence of at least one bacteriological contamination of drinking water was 52.26% (95%CI: 39.09–65.43), with extreme heterogeneity (I^2^ = 99.7%; p-value < 0.001) (Fig 2). Specifically among the type of bacteriological contaminant, the pooled prevalence of total coliform in drinking water in Ethiopia was found to be 49.55% (95% CI: 34.88–64.23). Extreme heterogeneity was observed among the included studies (I^2^ = 99.7%, p-value <0.001), random-effects model was carried out to determine the pooled prevalence of bacteriological contamination of drinking water in Ethiopia (Fig 3). The pooled prevalence of fecal coliform contamination of drinking water in Ethiopia was 44.27% (95%CI: 34.36–54.19) (Fig 4). Among different types of fecal coliform indicators, *E*. *coli* was the most prevalent in Ethiopian drinking water. This meta-analysis showed that the pooled prevalence of *E*.*coli* in drinking water was 31.35% (95% CI: 19.17–43.52) (Fig 5).

### Sanitary risk level of drinking water

Eight studies [25,28–30,32,33,44,48] were included to determine the sanitary risk level of drinking water sources. The meta-analysis showed more than one in seven water sources were at a very high sanitary risk level (14.13%; 95%CI: 7.64–20.63), with statistically significant heterogeneity (I^2^ = 94.1%, p< 0.001). Meta-analysis revealed that 17.8% and 24.6% of the sample water source had high and moderate sanitary risk levels, respectively, with extreme heterogeneity (Table 2).

**Table 2:** The pooled prevalence of sanitary risk level of drinking water sources in Ethiopia, 2024.

Risk level	Number of studies	Pooled prevalence (%)	Heterogeneity	
			I^2^	P-value
No risk	4	24.89(4.00–45.79)	95.8%	<0.001
Low risk	6	25.67(12.28–39.6)	95.7%	<0.001
Moderate risk	7	26.38(12.68–40.1)	96.9%	<0.001
High risk	8	17.8(13.17–22.43)	72.4%	<0.001
Very high risk	6	14.13(7.64–20.63)	94.1%	<0.001

### Publication bias assessment

**Fig 6:**
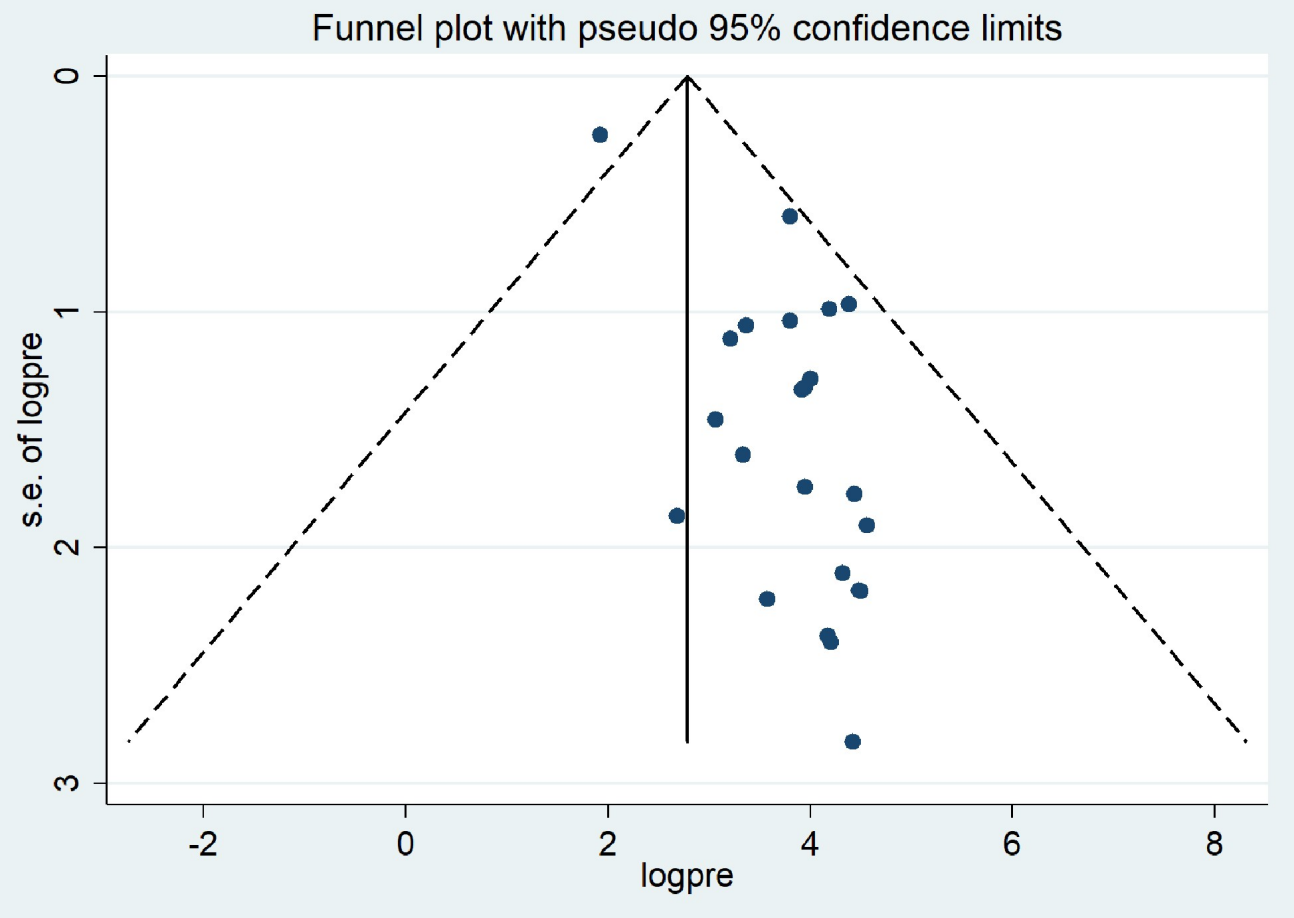
Funnel plot of the pooled prevalence of bacteriological quality of drinking water in Ethiopia, 2024.

**Fig 7:**
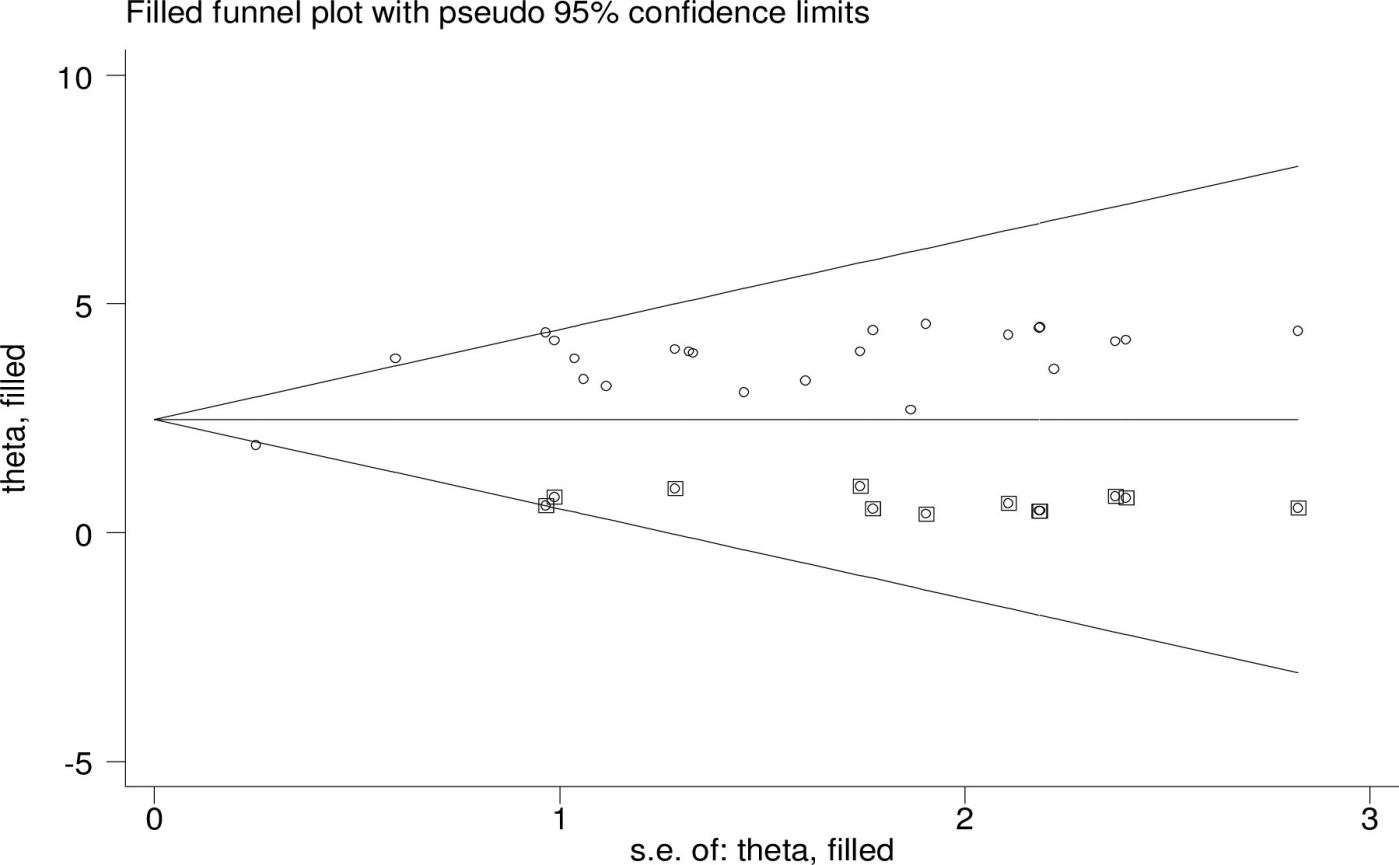
‘Trim and fill’ analysis funnel plot.

A funnel plot analysis revealed evidence of publication bias among the studies included in the meta-analysis (Fig 6). Further, the statistical Egger regression test also revealed the significant presence of publication bias (p < 0.001). To identify the source of this publication bias, Duval and Tweedie’s ‘trim and fill’ was carried out and the finding revealed significant variation in the newly estimated pooled odds ratio, represented as the adjusted point estimate [OR = 2.79, (95% CI: 2.27–3.31)] as compared to the initial or observed point estimate [OR = 3.45, (95% CI: 2.89–4.01)] (Fig 7).

### Sensitivity analysis

**Fig 8:**
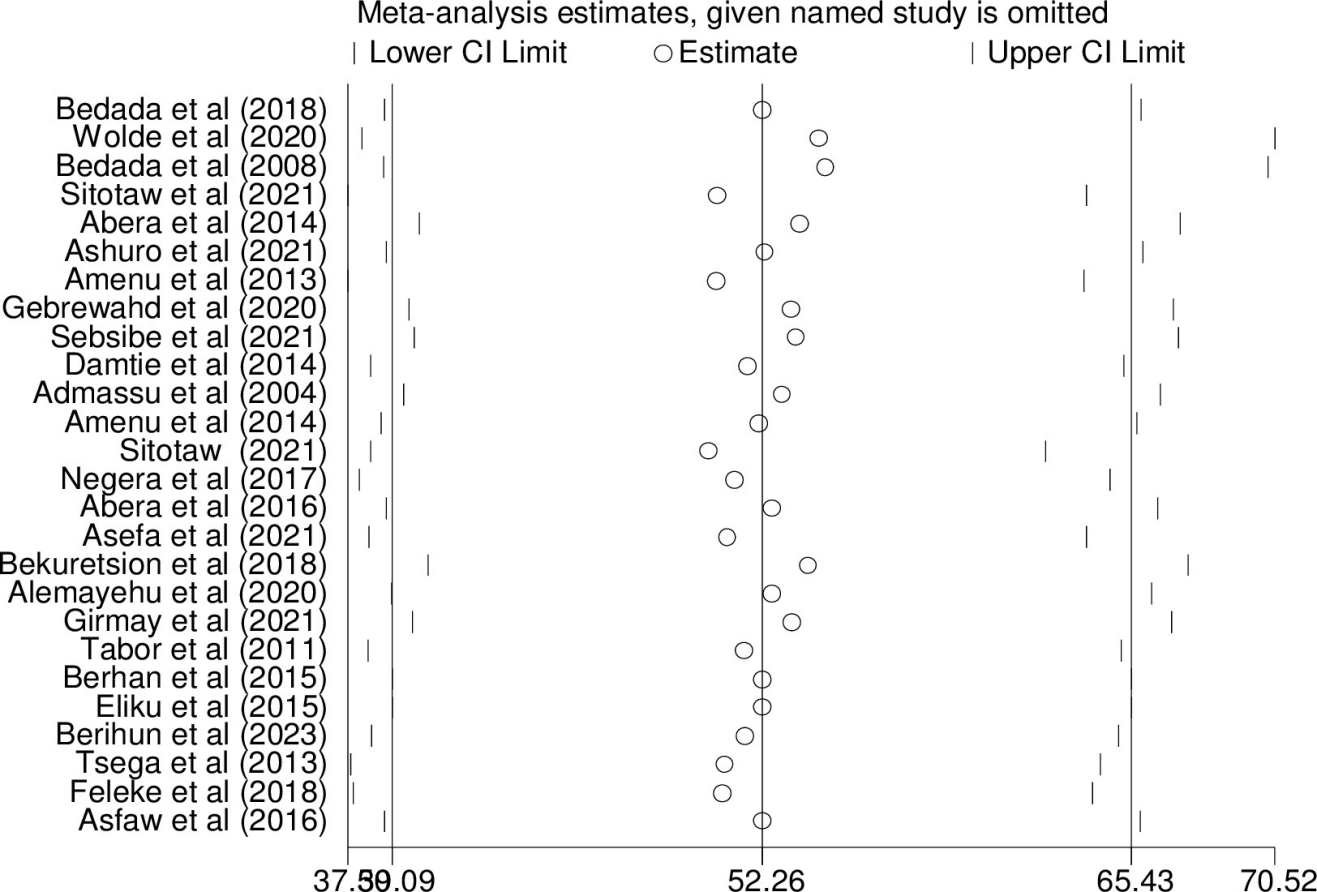
Sensitivity analysis of the pooled prevalence of bacteriological quality of drinking water in Ethiopia, 2024.

To determine the impact of individual studies on the overall pooled estimate, sensitivity analysis was carried out. The finding indicated that no single study exerted a significant effect (Fig 8).

### Meta-regression and subgroup analysis

**Fig 9:**
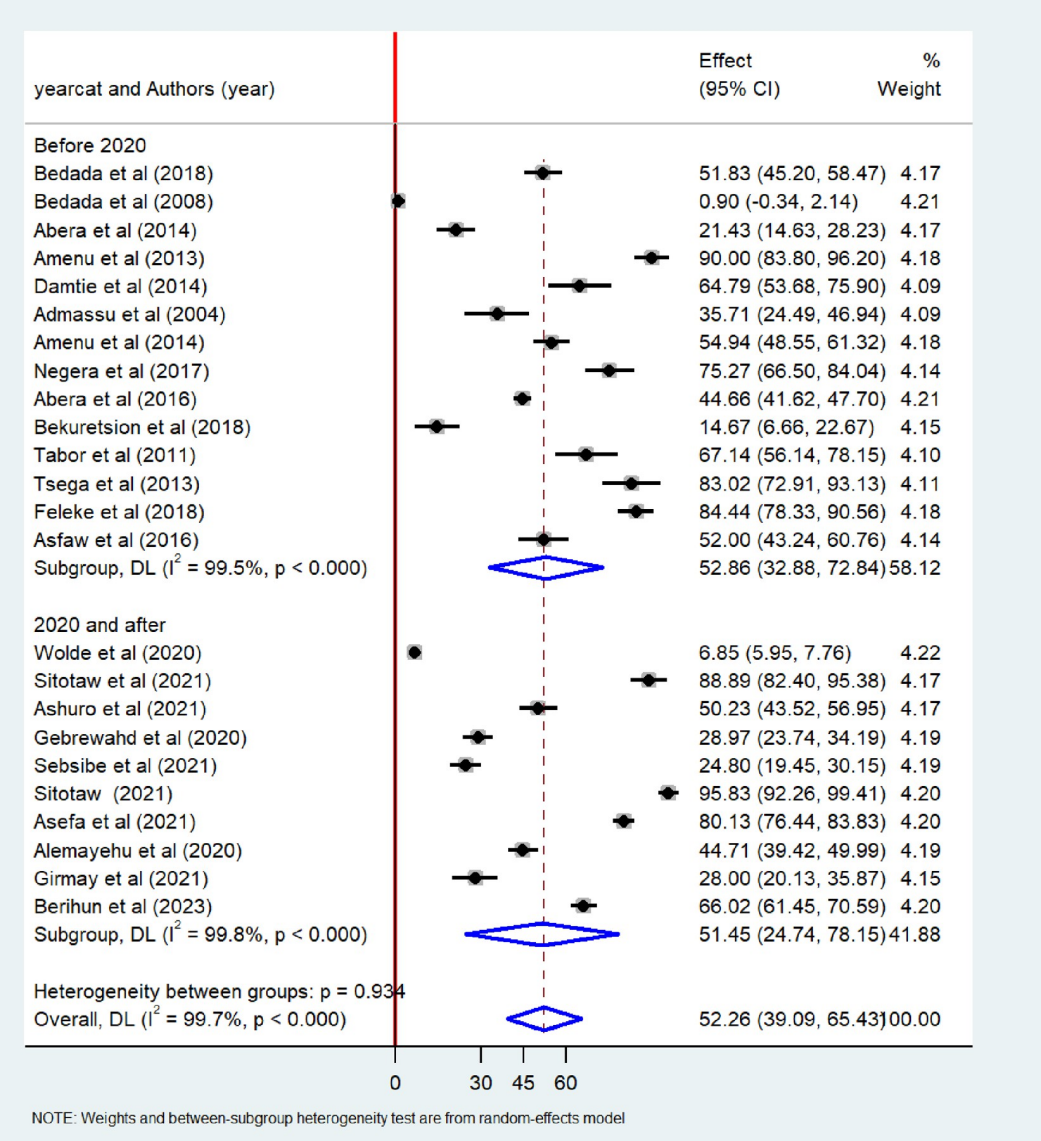
Subgroup analysis by year of publication of the pooled prevalence of bacteriological quality of drinking water in Ethiopia, 2024.

**Fig 10:**
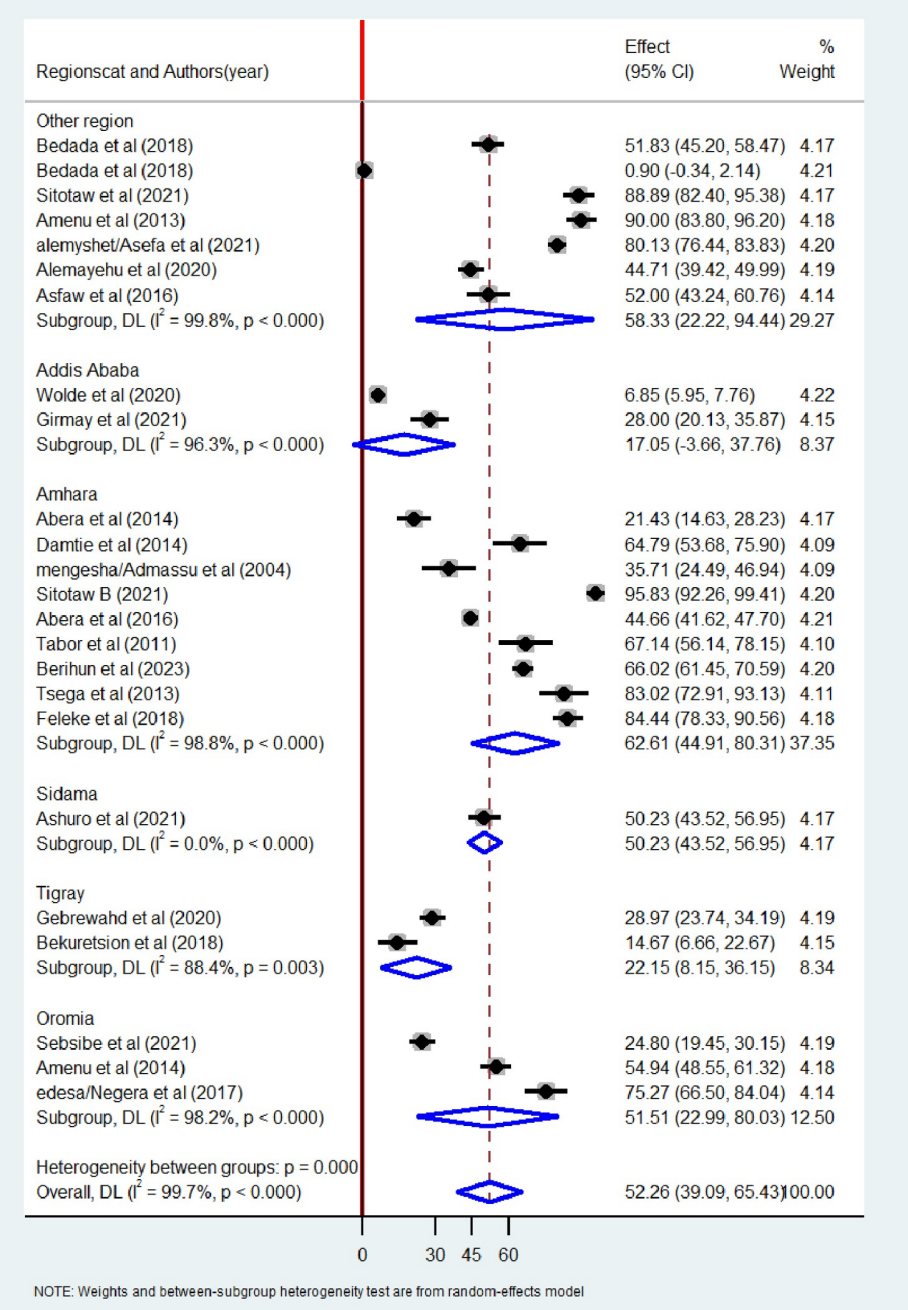
Subgroup analysis by region the pooled prevalence of bacteriological quality of drinking water in Ethiopia, 2024.

To identify the source of heterogeneity among the included studies, sub-group analysis was employed based on years of publication, regions and sample size of the study. The pooled prevalence of bacteriological quality of drinking water was found to be higher among studies conducted 2020 and after (52.86%; 95%CI: 32.88–72.84) than studies conducted before 2020 (51.45%; 95% CI: 24.74–78.15), with significant heterogeneity (I^2^ = 99.8%, p< 0.001) (Fig 9). When categorized regions, the highest prevalence of bacteriological contamination of drinking water was observed in studies conducted in the Amhara region (62.61%; 95%CI: 44.49–80.31) as compared to studies conducted in Oromia, Addis Ababa, Tigray, and other regions (Fig 10).

**Fig 11:**
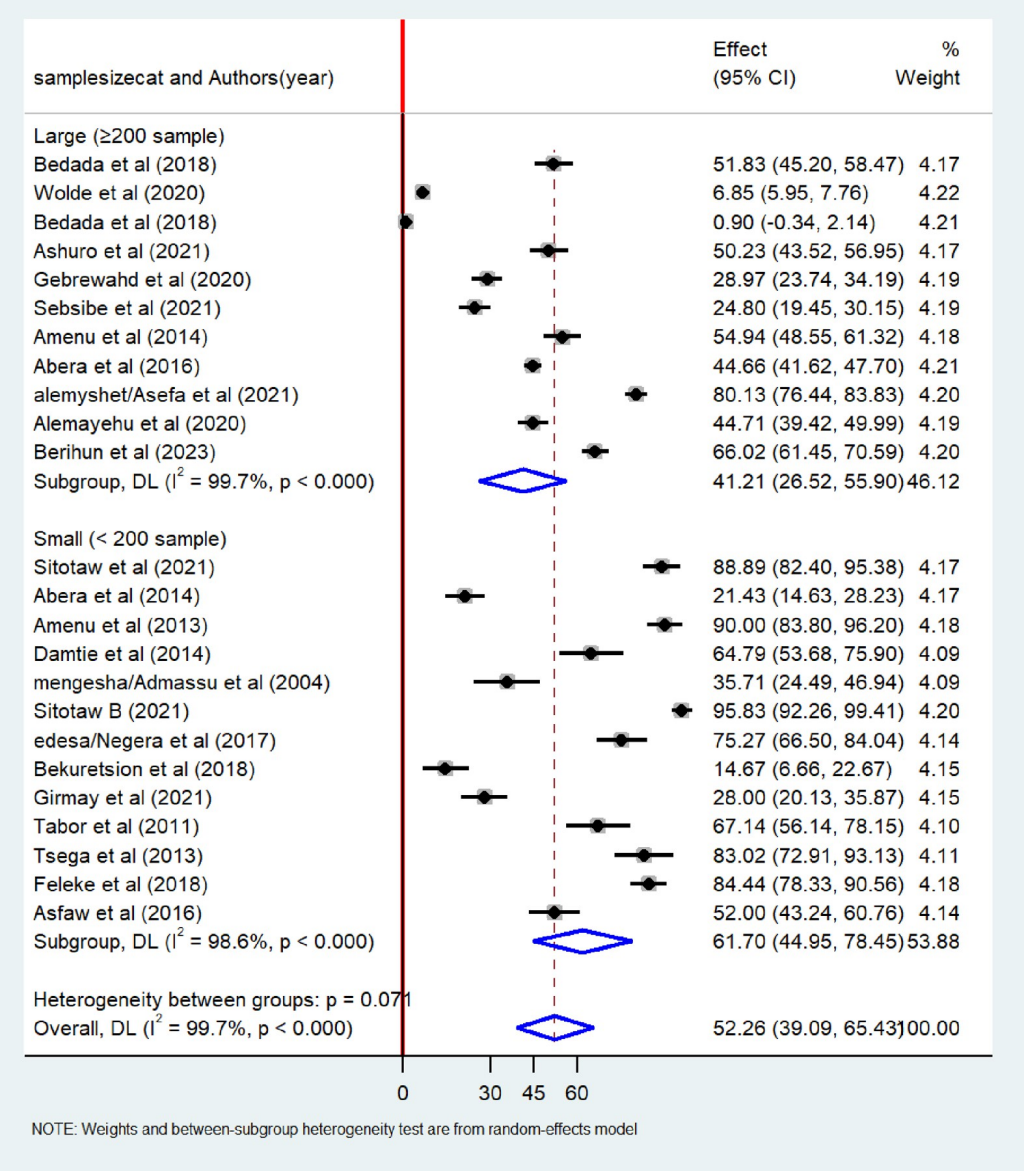
Subgroup analysis by region of the pooled prevalence of bacteriological quality of drinking water in Ethiopia, 2024.

Similarly, regarding the sample size of the study, the highest pooled of bacteriological contamination was observed among studies conducted with small sample size (61.70%; 95% CI: 44.95–78.45) than studies conducted with large sample size (41.21%; 95%CI: 26.52–55.90) (Fig 11). To identify the source of heterogeneity, a univariate meta-regression model was also employed based on the publication year, region and sample size of the study. The finding indicated that none of these variables demonstrated statistical significance (Table 3).

**Table 3:** Univariate meta-regression analysis to identify factors associated with the heterogeneity of the prevalence of bacteriological quality of drinking water in Ethiopia, 2024.

Variables	Coefficient	p-value
Year of publication	.905663	0.349
Sample size	2.603609	0.068
Study setting (region)	.4944022	0.221

### Factors associated with bacteriological quality of drinking water

Six studies [19,20,26,30,47,48] were included to identify factors associated with bacteriological quality of drinking water. The association between the presence of animal dung around water sources and the bacteriological quality of drinking water was examined based on the findings of two studies [19,48]. Two of the included studies had a positive association. Results from the random-effects model showed a non-significant association between the presence of animal dung around water sources with bacteriological quality of drinking water (OR = 7.88; 95%CI: -6.2–21.96). Two studies [19,48] were included to determine the association between the absence of fencing and the bacteriological quality of drinking water and two of them were positively associated. However, findings from the random-effects model revealed that there was a non-significant association between absences of fencing and bacteriological quality of drinking water (OR = 7.03; 95%CI:-11.45–25.52) (Table 4).

**Table 4:** The pooled factors association with bacteriological contamination of drinking water in Ethiopia, 2024.

Variables	Number of studies	OR (95% CI)	Heterogeneity	
			I^2^	p-value
presence of animal dung around the water source	2	7.88 (-6.2–21.96)	0.0%	0.995
Absence of fencing	2	7.03 (-11.45–25.52)	0.0%	0.983
Drawing using pocket/dipping	3	4.52 (1.71–7.34)	0.0%	0.002
Absence of water treatment	3	3.3 (1.28–5.32)	0.0%	0.003
Presence of pollution less than 10m radius	2	2.67 (-4.42–9.77)	0.0%	0.448

**Fig 12:**
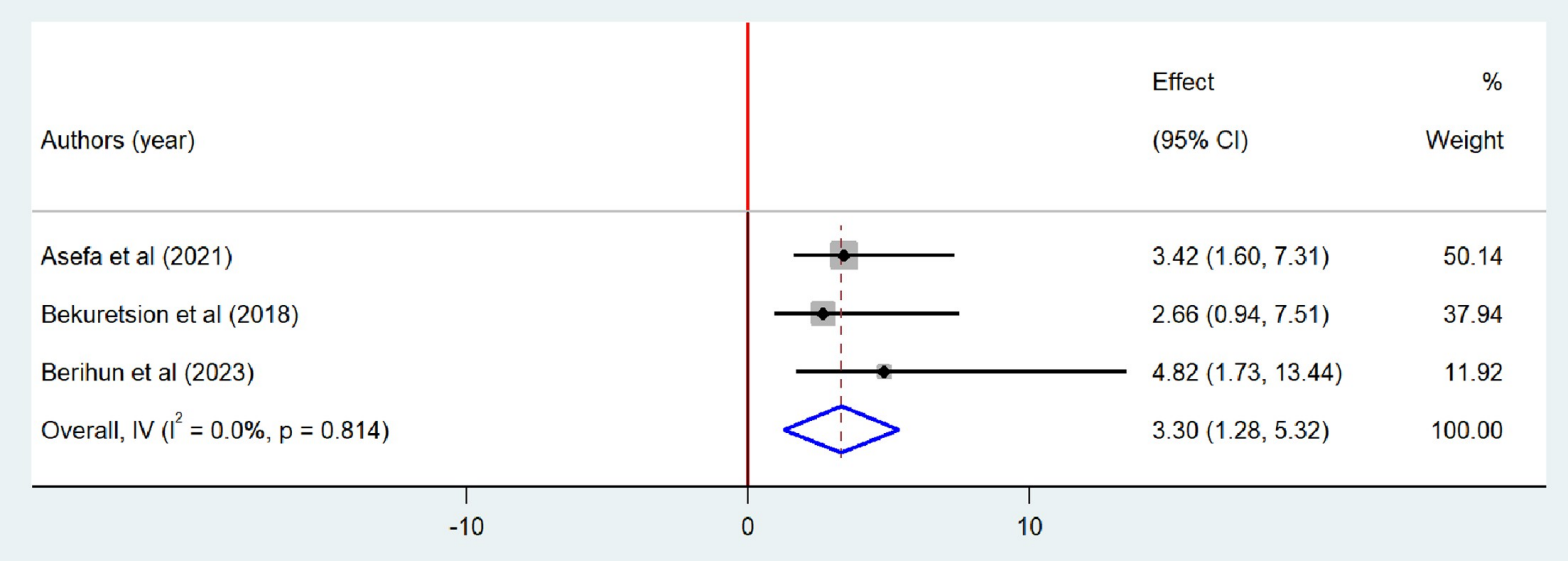
Forest plot of odds ratio for the association between absence of household level water treatment and bacteriological contamination of drinking water in Ethiopia, 2024.

A total of three studies [20,26,47] were included to determine the association between the absence of household-level water treatment and bacteriological contamination. Two of the included studies had a positive association [20,26] while a negative association in other studies [47]. The finding from the meta-analysis showed that the odds of bacteriological contamination of drinking water were three times higher in the absence of drinking water treatment than counterparts (OR = 3.3; 95%CI: 1.28–5.32) (Fig 12).

The association between the presence of pollution within less than a 10m radius of drinking water and bacteriological contamination of drinking water was examined based on the results of two studies [19,48]. One of the included studies had a positive association while negative in other studies. However, the finding from the meta-analysis showed that there was a non-significant association between the presence of pollution less than a 10m radius from drinking water and bacteriological contaminations of drinking water (OR = 2.67; 95% CI: -4.42–9.77) (Table 4).

**Fig 13:**
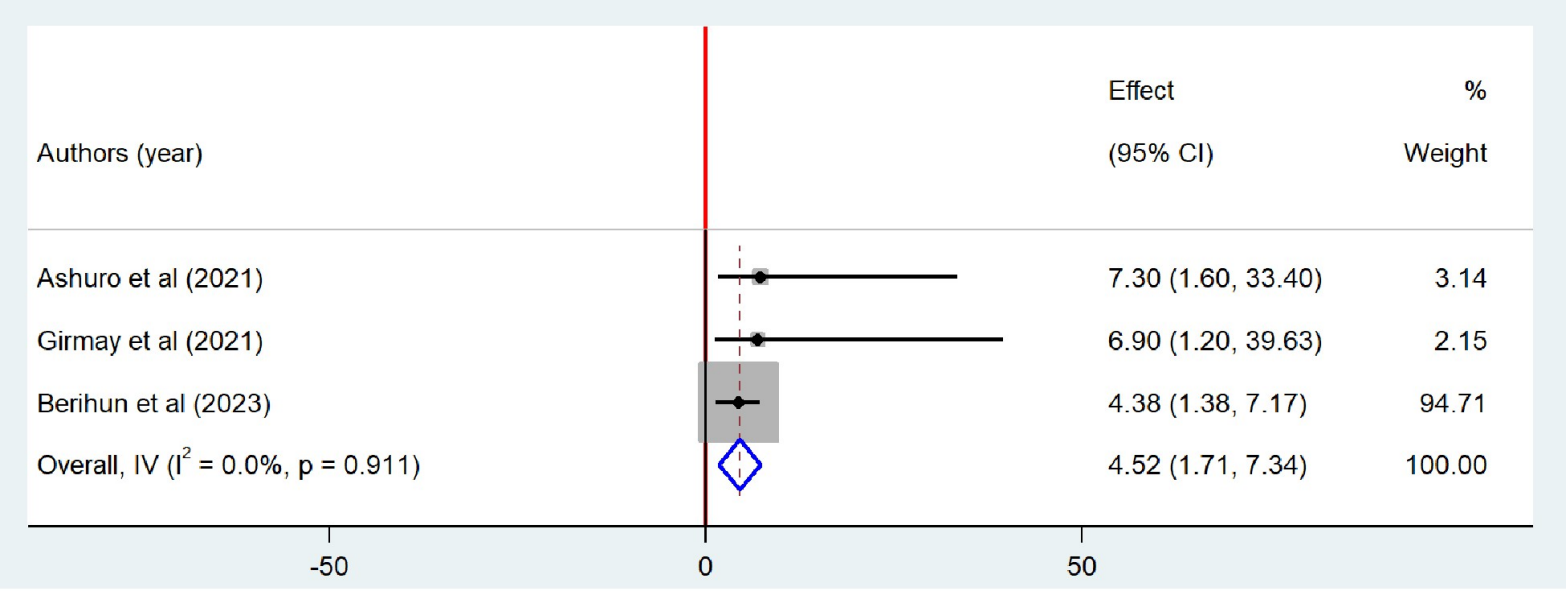
Forest plot of odds ratio for the association between drawing water using dipping and bacteriological contamination of drinking water in Ethiopia, 2024.

Similarly, three studies [19,26,30] were included to determine the association between drawing the water from the source using dipping and bacteriological contamination of drinking water. All of the included studies had a positive association. The finding from the random-effect model indicates that there was a significant association between drawing water using dipping and bacteriological quality of drinking water (OR = 4.52 (1.71–7.34) (Fig 13).

## Discussion

Drinking contaminated water remains a persistent public health problem in resource-limited countries including Ethiopia where access to safe drinking water is challenging [50]. The current meta-analysis revealed that 49.55% (95%CI: 34.88–64.23) of drinking water samples were contaminated with total coliforms, and 44.27% (95%CI: 34.36–54.19) were contaminated with fecal coliforms, which is lower than studies conducted in Ethiopia (72.6%) [51], Ghana (63%) [52] and Jimma (80%) [53]. On the other hand, the prevalence of fecal coliform in these findings was higher than in studies conducted in Kolladiba town (33%) [54], Nekemte town (37%) [55], Uganda (8.7%) [56], and Kenya (17.3%) [57]. This difference in bacteriological concentration might be due to the use of an unimproved water source in the study area rather than an improved water source. Unimproved drinking water sources are a major contributing factor to bacteriological contamination. These unimproved water sources are more susceptible to microbial contamination compared to improved water sources. Another possible reason could be poor hygiene and sanitation practices around water sources, which can cause bacteriological contamination of drinking water. The methods of water sample analysis could be another possible reason for the discrepancies in the prevalence reported by the studies. The current finding failed to comply with the world health organization and Ethiopian standard guidelines for drinking water quality (10CFU/ml for total coliform and 0 CFU/ml for fecal coliform and *E*.*coli*) [58,59], which call for urgent intervention to protect the health of the community.

The pooled prevalence of bacteriological contamination (*E*. *coli*) of drinking water was found to be 31.35%. This finding was consistent with a study conducted in Addis Ababa city (33%) [60]. On the other hand, *E*.*coli* concentration level in this meta-analysis was higher than studies done in Kisii Town, Kenya (17.5%) [57], South Darfur, Sudan (4.58%) [61] and lower than the evidence from a systematic review conducted in Africa (53%) [62] and Nekemte town (37%) [55]. The observed discrepancies in bacteriological contamination levels could be attributed to factors such as variations in the effectiveness of drinking water treatment technologies, deterioration of source water quality, failures in treatment processes, inadequate disinfection practices, and instances of cross-contamination within the distribution system.

Evidence from the meta-analysis indicated that the pooled prevalence of drinking water sources with a very high-risk and moderate sanitary risk score was found to be 14.13% (95% CI: 7.64–20.63) and 26.38% (95% CI: 12.68–40.1) respectively, which indicate the majority of drinking water source in Ethiopia, are unfit for drinking. This finding was consistent with a study conducted in north Gondar [51]. This finding was also supported by a study conducted in the south Gondar zone, which indicated that 29.8% of drinking water sources fell into the high microbial health risk category [63]. This finding indicates that the majority of drinking water in Ethiopia is beyond Ethiopian drinking water guidelines, which could endanger public health. This high sanitary risk level of water sources might be due to the majority of the Ethiopian population, especially in rural areas, relying on unimproved water sources such as unprotected wells, springs, or surface water bodies, which are more susceptible to fecal contamination from human or animal waste.

The absence of household-level water treatment was identified as a statistically significant factor for bacteriological contamination of drinking water. The meta-analysis indicates that the odds of the bacteriological quality of drinking water were three times higher among those who did not treat water at the household level compared to those who treated water at the household level. This finding was in agreement with studies conducted in Ethiopia [64] and Kenya [65]. In other words, Household-level water treatment plays a crucial role in reducing total and fecal coliform contamination in drinking water. Point-of-use treatment can effectively eliminate microorganisms, improving water quality. Furthermore, using disinfectants like chlorine during treatment can minimize the risk of recontamination.

Drawing water using dipping methods was four times more likely to result in bacteriological contamination compared to other methods of drawing drinking water, which is consistent with previous studies done in different parts of Kenya [57,65]. This indicated that withdrawing drinking water using the dipping methods could increase the risk of bacteriological contamination. The poor hygienic practices of drinking water handlers might be the possible reason for drinking water contamination [66].

### Strengths and limitations of the study

One of the strengths of the current meta-analysis is that it follows the updated Preferred Reporting Items for Systematic Reviews and Meta-Analyses (PRISMA) guidelines. Due to the lack of a comprehensive systematic review and meta-analysis, we compare the current findings with point prevalence. Only eight studies were included to determine the sanitary risk level of drinking water, which does not provide a clear indication of the overall sanitary risk level of drinking water in Ethiopia. Therefore, future studies should be conducted to assess the overall sanitary risk level of drinking water.

## Conclusion

We found that half of the water samples were contaminated with bacteria, and failed to comply with WHO guidelines and Ethiopian standard guidelines for drinking water, rendering them unfit for human consumption. The results from this meta-analysis showed that three out of eleven water sources were at a moderate sanitary risk level, and one out of seven water sources was at a high sanitary risk level, which could increase the risk of infectious disease in the country unless effective intervention is not taken place. Therefore, the water supply and sewerage authority should implement regular monitoring and inspection of drinking water quality. Additionally, federal and regional health offices should enhance health education programs focusing on water, sanitation, hygiene practices, household-level water treatment, and safe storage and handling of drinking water to improve point-of-use water quality.

## Supporting information

S1 File PRISMA-2020 checklist. (DOCX)

S2 File Search strategies. (DOCX)

S3 File Extracted data. (XLSX)

S4 File JBI Quality assessment. (DOCX)

## Abbreviations

JBI: Joana Briggs Institute
MF: Membrane Filtration
MPN: Most Probable Number
PRISMA: Systematic Reviews and Meta-Analysis
SDGs: Sustainable Development Goals
SNNPR: Southern Nation, Nationalities and People Region
WASH: water, sanitation, and hygiene
WHO: World Health Organization
