# Supplementary material for: Bacteriological quality of drinking water and its associated factors in Ethiopia: A systematic review and meta-analysis
Source: PLoS One. 2025 Jan 3;20(1):e0310731. doi: 10.1371/journal.pone.0310731 (PMC11698375; doi:10.1371/journal.pone.0310731)
Supplement: S2 File — (DOCX) [file pone.0310731.s002.docx]

| Mesh heading | Combination | Number of article | Last search date | Electronic data base |
| --- | --- | --- | --- | --- |
|  | ((((((((bacteriological quality [tiab]) OR (total coliform [tiab])) OR (fecal coliform[tiab])) OR (Thermotolerant[tiab])) OR ("Micriobial"[All Fields] AND "quality"[Title/Abstract])) AND (water quality[tiab])) OR (water sourcse[tiab])) OR (surface water [tiab])) AND (Ethiopia [tiab]) | 168 | 8/8/2024 | PubMed |
|  | ((((((((((((((bacteriological quality) OR (microbial quality)) OR (total coliform)) OR (fecal coliform)) OR (thermotolerant)) AND (drinking water)) OR (water)) OR (water source)) OR (protected water source)) OR (unprotected water source)) OR (tap)) AND (factors)) OR (associated factors)) OR (determinant factors)) AND (Ethiopia) |  |  |  |
|  | ((bacteriological quality) OR (microbial quality) OR (total coliform) OR (fecal coliform) OR (thermotolerant)) AND ((drinking water) OR (water) OR (water source) OR (protected water) OR (unprotected water source) OR (tap)) AND ((associated factors) OR (factors) OR (determinant factors)) AND (Ethiopia) | 52 | 10/8/2024 | Hinari |
|  | Bacteriological quality of drinking and its associated factors in Ethiopia | 102 | 10/8/2024 | Science Direct |
|  | Bacteriological quality of drinking and its associated factors in Ethiopia | 181 | 10/8/2024 | Google scholar |
|  | Bacteriological quality of drinking and its associated factors in Ethiopia | 31 | 10/8/2024 | African journal online |
| **S/No** | **Tittle of the study** | **Reason of exclusion** | **Remark** | |
|  | Kassegne AB, Leta S. Assessment of physicochemical and bacteriological water quality of drinking water in Ankober district, Amhara region, Ethiopia. Cogent Environmental Science. 2020 Jan 1;6(1):1791461. | Did not report outcome of the interest for the meta-analysis. |  | |
|  | Gizachew M, Admasie A, Wegi C, Assefa E. Bacteriological contamination of drinking water supply from protected water sources to point of use and water handling practices among beneficiary households of boloso sore woreda, wolaita zone, Ethiopia. International Journal of Microbiology. 2020;2020(1):5340202. | Did not report outcome of the interest for the meta-analysis. |  | |
|  | Yasin M, Ketema T, Bacha K. Physico-chemical and bacteriological quality of drinking water of different sources, Jimma zone, Southwest Ethiopia. BMC research notes. 2015 Dec;8:1-3. | Did not report outcome of the interest for the meta-analysis. |  | |
|  | Siyum D, Woyessa D. Assessment of bacteriological quality and traditional treatment methods of water-borne diseases among well water users in Jimma Town, South West Ethiopia. ARPN J Ag & Bio Sci. 2013;8:477-86. | Failed due to JBI quality assessment |  | |
|  | Abera S, Zeyinudin A, Kebede B, Deribew A, Ali S, Zemene E. Bacteriological analysis of drinking water sources. African Journal of Microbiology Research. 2011 Sep 16;5(18):2638-41. | Failed due to JBI quality assessment |  | |
|  | Garoma B, Kenasa G, Jida M. Drinking water quality test of shambu town (Ethiopia) from source to household taps using some physico-chemical and biological parameters. Research & Reviews: Journal of Ecology and Environmental Sciences. 2018;6(4):82-8. | Failed due to JBI quality assessment |  | |
