# Supplementary material for: Bacteriological quality of drinking water and its associated factors in Ethiopia: A systematic review and meta-analysis
Source: PLoS One. 2025 Jan 3;20(1):e0310731. doi: 10.1371/journal.pone.0310731 (PMC11698375; doi:10.1371/journal.pone.0310731)
Supplement: S4 File — (DOCX) [file pone.0310731.s004.docx]

**Results of JBI Quality Assessment**

| Studies | Clear eligibility criteria | Description of study subject and study setting | Valid and reliable method to measure the exposure | Standard criteria used for measurement of the condition | Identification of confounding factors | Develop of strategies to deal with confounding factors | Valid and reliable method to measured outcomes | Appropriate statistical analysis | Quality score (%) |
| --- | --- | --- | --- | --- | --- | --- | --- | --- | --- |
| Bedada et al | Yes | Yes | Yes | Yes | Yes | No | Yes | Yes | 87.5 |
| Wolde et al | Yes | Yes | Yes | Yes | No | No | Yes | Yes | 75.0 |
| Bedada et al | Yes | Yes | Yes | No | Yes | No | Yes | Yes | 87.5 |
| Sitotaw et al | Yes | Yes | Yes | Yes | Yes | No | No | Yes | 75.0 |
| Abera et al | Yes | Yes | Yes | No | Yes | No | Yes | Yes | 75.0 |
| Ashuro et al | Yes | Yes | Yes | Yes | No | No | Yes | Yes | 75.0 |
| Amenu et al | Yes | Yes | Yes | No | No | No | Yes | Yes | 62.5 |
| Gebrewahd et al | Yes | Yes | Yes | Yes | No | No | Yes | Yes | 75.0 |
| Sebsibe et al | Yes | Yes | Yes | No | Yes | No | Yes | Yes | 75.0 |
| Damtie et al | Yes | Yes | Yes | Yes | Yes | No | Yes | Yes | 87.5 |
| Admassu et al | Yes | Yes | Yes | Yes | Yes | No | Yes | Yes | 87.5 |
| Amenu et al | Yes | Yes | No | Yes | No | No | Yes | Yes | 62.5 |
| Sitotaw | Yes | Yes | Yes | Yes | No | No | Yes | Yes | 75.0 |
| Negera et al | Yes | Yes | Yes | No | No | No | Yes | Yes | 62.5 |
| Abera et al | Yes | Yes | No | Yes | No | Yes | Yes | Yes | 75.0 |
| Asefa et al | Yes | Yes | No | Yes | No | Yes | Yes | Yes | 75.0 |
| Bekuretsion et al | Yes | Yes | Yes | Yes | Yes | No | Yes | Yes | 87.5 |
| Alemayehu et al | Yes | Yes | Yes | Yes | Yes | No | Yes | Yes | 87.5 |
| Girmay et al | Yes | Yes | Yes | No | No | No | Yes | Yes | 62.5 |
| Tabor et al | Yes | Yes | Yes | Yes | No | No | Yes | Yes | 75.0 |
| Berhanu et al | Yes | Yes | Yes | Yes | Yes | No | Yes | Yes | 87.5 |
| Eliku et al | No | Yes | Yes | Yes | No | No | Yes | Yes | 75.0 |
| Berihun et al | Yes | Yes | Yes | No | No | Yes | Yes | Yes | 75.0 |
| Tsega et al | Yes | Yes | Yes | No | No | Yes | Yes | Yes | 75.0 |
| Feleke et al | Yes | Yes | Yes | No | No | Yes | Yes | Yes | 75.0 |
| Asfaw et al | Yes | Yes | Yes | No | No | Yes | Yes | Yes | 75.0 |
